# Development of the New Sensor Based on Functionalized Carbon Nanomaterials for Promethazine Hydrochloride Determination

**DOI:** 10.3390/s23052641

**Published:** 2023-02-27

**Authors:** Mirela Samardžić, Mateja Peršić, Aleksandar Széchenyi, Marija Jozanović, Iva Pukleš, Mateja Budetić

**Affiliations:** 1 Department of Chemistry, Josip Juraj Strossmayer University of Osijek, Cara Hadrijana 8/A, 31000 Osijek, Croatia; 2 Doctoral School of Chemistry, University of Pécs, Ifjúság útja, 7624 Pécs, Hungary

**Keywords:** promethazine hydrochloride, solid-contact sensor, potentiometry

## Abstract

Promethazine hydrochloride (PM) is a widely used drug so its determination is important. Solid-contact potentiometric sensors could be an appropriate solution for that purpose due to their analytical properties. The aim of this research was to develop solid-contact sensor for potentiometric determination of PM. It had a liquid membrane containing hybrid sensing material based on functionalized carbon nanomaterials and PM ions. The membrane composition for the new PM sensor was optimized by varying different membrane plasticizers and the content of the sensing material. The plasticizer was selected based on calculations of Hansen solubility parameters (HSP) and experimental data. The best analytical performances were obtained using a sensor with 2-nitrophenyl phenyl ether (NPPE) as the plasticizer and 4% of the sensing material. It had a Nernstian slope (59.4 mV/decade of activity), a wide working range (6.2 × 10^−7^ M–5.0 × 10^−3^ M), a low limit of detection (1.5 × 10^−7^ M), fast response time (6 s), low signal drift (−1.2 mV/h), and good selectivity. The working pH range of the sensor was between 2 and 7. The new PM sensor was successfully used for accurate PM determination in a pure aqueous PM solution and pharmaceutical products. For that purpose, the Gran method and potentiometric titration were used.

## 1. Introduction

Promethazine hydrochloride (PM) or N,N-dimethyl-1-phenothiazin-10-yl-propan-2-amine hydrochloride is a widely used phenothiazine derivative. It has antihistaminic, anti-emetic, sedative, antipsychotic, and analgesic properties, so it is mainly used to treat mental illness, prevent motion sickness, and as an allergy medication [1,2]. Additionally, it can be used in a broad range of health conditions such as asthma, pneumonia, respiratory tract infections, hemorrhoids, intracranial hypertension, and neoplastic disorders, or as a melanogenesis-suppressing agent, skin-aging prevention agent, and sperm-killing agent [3]. Recently, PM has been recommended in the treatment of COVID-19 [4,5]. However, PM can cause some serious adverse effects, including severe breathing problems, depression of the central nervous system, cardiac problems, endocrine disorders, gastrointestinal effects, immunoallergic reactions, venous thrombosis, sudden infant death syndrome, tissue damage, etc. [3,6]. Even U.S. Food and Drug Administration (FDA) issued an alert in 2006, with notification that PM causes fatal respiratory depression in children younger than two years [7], and in 2009, considering the risk of severe tissue damage after intravenous injection [8]. The widespread nonmedical abuse of PM has also been observed in last few decades [9,10]. Due to its wide use, the monitoring of PM is very important. PM can be determined using different methods, including liquid chromatography-tandem mass spectrometry [11], high performance liquid chromatography [12], gas chromatography [13], colorimetry [14], spectrophotometry [15], turbidimetry [16], capillary zone electrophoresis [17], and chemiluminescence [18]. Although these methods allow accurate PM determination, most require expensive instrumentation, tedious and complex sample preparations and measuring procedures, and a large quantity of organic solvents. On the contrary, electrochemical methods such as voltammetry [1,2] and potentiometry [19,20], available in most analytical laboratories, proved to be simpler but also accurate, sensitive, and selective. Trends in development of voltammetric methods for PM determination include modification of glassy carbon or screen-printed electrodes using functional materials [2,21] and nanomaterials [1,22]. Ion-selective electrodes (ISEs), used as sensors in potentiometry, are easy to develop, and their membranes are easy to modify. It is important because modifying the membrane composition leads to the development of the sensor with better analytical properties (Nernstian slope, a lower limit of detection (LOD), wider measuring range, better selectivity, etc.). The membrane of the ISE usually consists of an ionophore, polyvinyl chloride (PVC), and plasticizer. As an ionophore, which is the sensing element in the membrane, ion pairs [23], cyclodextrins [20], metal-organic frameworks [24], or functionalized nanomaterials [25] can be used. The most commonly used sensing materials in potentiometric PM sensors are ion pairs: PM and tetraphenylborate [19,23,26], PM and phosphomolybdate [27,28], and PM and phosphotungstic acid [29]. As well as the ionophore, the plasticizer also impacts the response of the ISE and its lifetime [30]. Considering the above, the type and percentage of the ionophore and type of plasticizer are usually varied during membrane modification.

Most of the potentiometric sensors for PM determination are conventional sensors with the liquid type of membrane and internal filling solution [23,26,27,28,29]. The drawback typical for these type of sensors is leaching of the sensor material which can contribute to poor analytical performance and reduced lifetime of the sensors. It can be overcome by constructing solid-contact electrodes without internal filling solution or by modifying the membrane composition [31]. Functionalized multi-walled carbon nanotubes (MWCNTs) proved to be convenient for that purpose [31].

This work describes the development of a new potentiometric sensor for PM determination. The research aimed to prepare a simple, accurate, and selective solid-contact sensor based on new hybrid sensing material with MWCNTs. The membrane composition was optimized using calculations and experiments, and MWCNTs were used for the first time in order to improve analytical performance of the new PM sensor. The potentiometric characterization and the applicability of the developed sensor in pharmaceutical products were demonstrated.

## 2. Materials and Methods

### 2.1. Sensor Preparation

The new sensing material (MWCNT−OSO_3_PM) was prepared by functionalization of MWCNTs (MWCNT−OH, 95+%, IoLiTec, Germany, o.d. of 20 to 30 nm and a length of 10 to 30 μm) with chlorosulfonic acid (Acros Organics B.V.B.A., Belgium) according to [31], and then mixing with the solution of PM (*c* = 1 × 10^−2^ M). PM solution was added dropwise until the formation of a flaky precipitate. After magnetic stirring for 2 h, the formed precipitate was washed with water and dried under low pressure in a water bath at 35 °C using a rotary evaporator.

The membrane of the sensor was prepared using MWCNT−OSO_3_PM, PVC (Fluka, Buchs, Switzerland), and plasticizer (*o*-nitrophenyl octyl ether (*o*-NPOE), dibutyl phthalate (DBP), 2-nitrophenyl phenyl ether (NPPE), dibutyl sebacate (DS), and bis(2-ethylhexyl) phthalate (DOP), all from Fluka, Switzerland). During modifications of the membrane, the PVC and plasticizer ratio was always 1:2, and the content of the sensing material was 2%, 4%, or 6%. The mass of the membrane was always 0.0916 g. Membrane components were dissolved in a mixture of 1 mL of tetrahydrofuran and 10 µL of dimethylformamide. The body of the sensor was SIMONA^®^ PVC-C CORZAN rod (SIMONA AG, Kirn, Germany) with 12 mm o.d. Drop-casting of the graphene layer (Gwent group, Pontypool, UK) on the spectral graphite core (o.d. 6 mm) of the sensor was followed by drop-casting of the liquid membrane on it. Before performing measurements, the new sensor was left to dry for 24 h at room temperature.

### 2.2. Reagents and Materials for Measurements

Racemic PM (Hungaropharma, Budapest, Hungary) was used to prepare the analyte. Analytical grade chemicals were used for the preparation of all salt solutions used as potential interferents, as well as for L(+)-ascorbic acid (GRAM-MOL, Zagreb, Croatia), glycine (Carlo Erba, Cornaredo, Italy), naproxen sodium (Thermo Scientific, Waltham, MA, USA), caffeine (Carlo Erba, Italy), HCl (Carlo Erba, Italy), and NaOH (GRAM-MOL, Croatia) solutions. Sodium tetraphenylborate (NaTPB, Fluka, Switzerland) was used as the titrant. All solutions were prepared using deionized water with a conductivity of 0.055 µS/cm. Atosil drops (20 mg/mL, Desitin Arzneimittel GmbH, Hamburg, Germany) and Promethazin-Neuraxpharm drops (20 mg/mL, Neuraxpharm Arzneimittel GmbH, Langenfeld, Germany) were used as real samples.

### 2.3. Apparatus

The Thermo Nicolet Avatar 380 FTIR with Smart Orbit Diamond ATR (Thermo Scientific, USA) was used for ATR-FTIR spectra recording. An ultrasonic bath (BANDELIN RK-100, Berlin, Germany) was used to prepare liquid membranes and solutions. The 794 Basic Titrino, 806 Exchange unit, 826 mobile pH meter, and 728 stirrer (all from Metrohm, Herisau, Switzerland) were used for measurements. Devices were controlled with Tiamo software (Metrohm, Switzerland) and in-house software.

### 2.4. Procedure

FTIR characterization was conceived by Thermo Nicolet 380 FTIR spectroscope using Smart Orbit diamond ATR sampling attachment. FTIR spectra were recorded with 4 cm^−1^ wavenumber resolution. In the range of 4000–400 cm^−1^, 100 scans were recorded and averaged. The baseline was taken under ambient conditions on a cleaned ATR diamond surface. Samples of MWCNT-OSO_3_H, PM, and MWCNT-OSO_3_PM, were dried in a vacuum desiccator for 24 h before the examination (*p* = 0.1 mbar).

For all measurements, the new sensor was used as the indicator electrode, and Ag/AgCl electrode (Metrohm, Switzerland) as the reference electrode. The measurement conditions were: no pH and ionic strength adjustment, room temperature, and magnetic stirring.

Every day before measurements, the sensor was left for 15 min in PM (*c* = 1.0 × 10^−2^ M) for conditioning. It was followed by calibration to check the sensor’s reliability.

Response measurements were performed by incrementally adding PM solution (*c* = 1.0 × 10^−2^ M and 5.0 × 10^−5^ M) to 20 mL of distilled water. For dynamic response measurements, PM (*c* = 5.0 × 10^−2^ M, 5.0 × 10^−3^ M, and 5.0 × 10^−4^ M) was incrementally added, every 30 s, to 50 mL of PM solution (*c* = 1.0 × 10^−7^ M). Signal drift was measured in 20 mL of PM solution (*c* = 4.0 × 10^−3^ M). The selectivity of the sensor was investigated using the fixed interference method [32], where PM solution (*c* = 4.0 × 10^−3^ M) was incrementally added to 20 mL of potential interference solution (*c* = 1.0 × 10^−2^ M). Finally, the influence of the pH value on the response of the sensor was investigated using NaOH and HCl solutions (*c* = 1.0 M, 1.0 × 10^−1^ M, and 1.0 × 10^−2^ M). Real samples were prepared by dissolving drops of medicines in distilled water. It was followed by PM determination using a titration of 25 mL of the sample with NaTPB (*c* = 4.0 × 10^−3^ M) and the Gran method, where nine increments of PM (*c* = 2.0 × 10^−3^ M) were added in 15 mL of the sample solution. The measuring devices were controlled with in-house software for all measurements except for titrations, where Tiamo software was used.

## 3. Results

### 3.1. ATR-FTIR Characterization of MWCNT−OSO_3_PM

**Figure 1:**
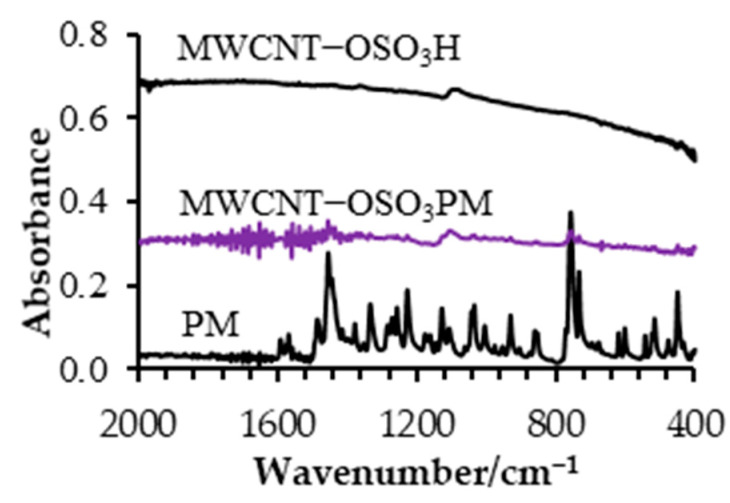
ATR−FTIR spectra of MWCNT−OSO_3_H, PM, and MWCNT−OSO_3_PM.

MWCNT−OSO_3_H was prepared and characterized by FTIR in previous study [31]. The FTIR spectrum of PM (Figure 1) shows all characteristic peaks for PM [33]. A peak at 449 cm^−1^ is assigned to the stretching modes of C-S bonds. Stretching modes of some C-N bonds are observed at 755 and 1230 cm^−1^, while peaks at 1100 and 1455 cm^−1^ are assigned to aromatic out-of-plane vibrations. These PM characteristic modes are observed in MWCNT−OSO_3_PM with small intensities, but they are not observed on the MWCNT−OSO_3_H sample, which is indirect proof of PM bonding to MWCNT−OSO_3_H. Due to the high IR absorbance of MWCNT, the small changes in FTIR spectra due to PM bonding cannot be identified or observed.

### 3.2. Response of the Sensor

The newly developed sensor is a solid-contact ISE. Its liquid membrane, which is responsible for the response of the sensor, contains MWCNTs covalently functionalized with a sulfate group and PM ion as an ionophore. The sensor has a potentiometric response to PM according to the Nernst equation:

(1) $$E=E^{0}+S\cdot loga_{PM^{+}}$$

where *E* is measured electrode potential, *E*^0^ is standard electrode potential, *S* is the slope of the sensor, and $a_{PM^{+}}$ is the activity of the PM ion. The Nernstian slope representing the theoretical slope for PM ion is 59.2 mV/decade of activity at 25 °C.

The MWCNT−OSO_3_PM in the sensor membrane dissociates according to the Equation (2):

(2) $$\mathrm{MWCNT}-{\mathrm{OSO}}_{3}\mathrm{PM}⇄\mathrm{MWCNT}-{\mathrm{OSO}}_{3}^{-}+{\mathrm{PM}}^{+}$$

MWCNTs are especially convenient to use as part of the ionophore considering their good electrical properties that makes MWCNT−OSO_3_^−^ sensitive to changes in a solution of the analyte. Additionally, their hydrophobicity and structure lead to the immobility of MWCNT−OSO_3_^−^ in the membrane and the formation of an electrostatic barrier for anions penetration. ${\mathrm{PM}}^{+}$ ions in the membrane close to the MWCNT−OSO_3_^−^ regulate their charge. As a result, the ion-to-electron transduction between the electrons in the MWCNT−OSO_3_^−^ wall and ${\mathrm{PM}}^{+}$ ions in the membrane occurs. The electrical double layer on MWCNT−OSO_3_^−^ near the interface between membrane and solution is related to the concentration of ${\mathrm{PM}}^{+}$ ions in solution. Subsequently, an electromotive force is generated [34,35,36]. The described type of sensor can be successfully applied for the potentiometric determination of PM.

### 3.3. Selection of the Plasticizer

The plasticizer is a predominant component of the ISE membrane. Its main function is to reduce the viscosity and improve the physical and mechanical properties of the membrane, thus improving the mobility inside the membrane. Additionally, it is well-known that its nature, especially the polarity, significantly impacts the ISE’s selectivity, slope, and LOD [30,37].

The plasticizer’s insolubility in water and mixability with the PVC matrix determines the physical properties of the membrane and the stability of the sensor. At the same time, its molecular compatibility with the analyte determines its sensing properties. Several theoretical methods can calculate molecular interaction properties. For the plasticizer selection, calculations of Hansen solubility parameters (HSP) were performed. HSP splits a liquid’s total cohesion energy into contributions from hydrogen bonding (δh), atomic dispersion (δd), and polar interactions (δp), which forms a 3D Hansen parameter space [38]. The logarithm of the partition coefficient (logP), logarithm of the solubility (logS), and HSP for plasticizers and PM were calculated by Hansen Solubility Parameters in Practice (HSPiP) software version 5.2.02., using simplified molecular-input line-entry system (SMILES) obtained from the open chemistry database PubChem. The calculated data are shown in Table 1. Materials with logP > 3 and logS < −1 are considered water-insoluble, which is the requirement that all selected plasticizers meet. The plasticizer’s affinity toward the analyte (PM) can be estimated from R_a_, the HSP distance of two materials in the 3D Hansen parameter space. It shows molecular similarity. The smallest value shows the largest similarity and likely the best solubility of PM in a given plasticizer. From this point of view, NPPE is predicted to be the best plasticizer for PM selective membranes.

Experimental measurements were also performed to select the best plasticizer for the new PM sensor. Responses of five ISEs containing five different plasticizers (DBP, *o*-NPOE, DS, NPPE, and DOP) were measured. All ISEs investigated contained 2% of sensor material (MWCNT−OSO_3_PM), and the weight ratio of plasticizer and PVC was 2:1. The response characteristics of ISEs towards PM were investigated in a concentration range between 2.5 × 10^−8^ M and 5.0 × 10^−3^ M. The results are presented in Table 2. The statistical data were based on five repeated measurements and calculated using linear regression analysis. The IUPAC recommendations [39] were used for the estimation of LOD. Regardless of the plasticizer used, all sensors revealed a sub-Nernstian response. However, the sensor with NPPE revealed the slope value closest to Nernstian (55.8 mV/decade of activity). The LOD was lowest for a sensor with DS as a plasticizer, but it was not selected for further investigation due to its lower slope value. Considering the results and theoretical calculations obtained using HSPiP, the sensor with NPPE as a plasticizer was chosen for additional research.

### 3.4. Optimization of the Sensor Material Content

**Figure 2:**
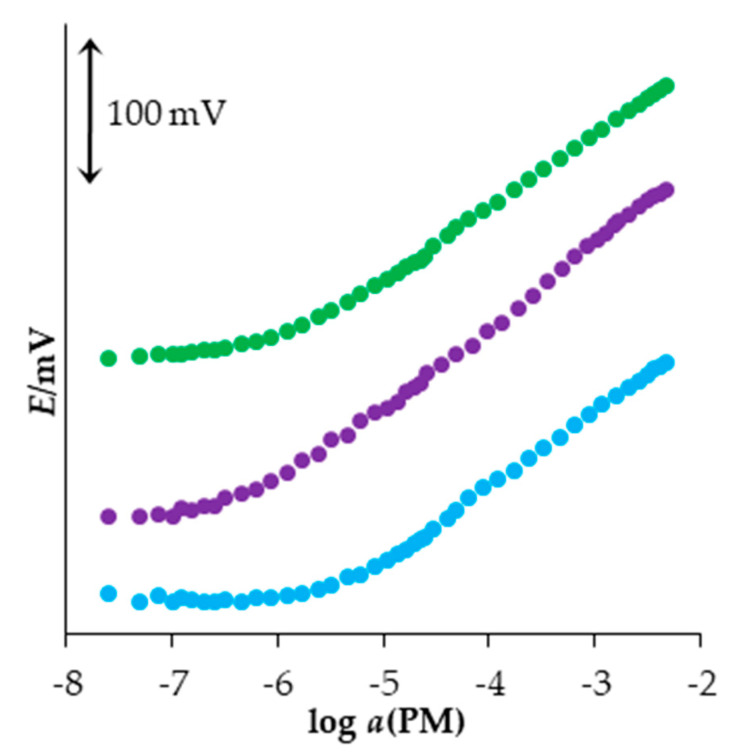
Responses to PM obtained using the PM sensors with different content of sensor material (● 2%, ● 4%, and ● 6%). Some curves are displaced vertically for clarity.

After selecting the most suitable plasticizer for the new PM sensor, the influence of the content of the sensor material on the response characteristics of the new sensor was investigated. It was based on the results of measuring the response to PM of three ISEs containing NPPE as a plasticizer and different content of MWCNT−OSO_3_PM (2%, 4%, and 6%). The weight ratio of the plasticizer and PVC was 2:1. The resulting response curves and their statistics, based on five repeated measurements, are shown in Figure 2 and Table 3.

It can be seen that the sensor with 4% of sensor material in its membrane exhibited the Nernstian slope (59.4 mV/decade of activity), while the other two sensors investigated exhibited the sub-Nernstian slope. Additionally, it has the lowest LOD and the widest measuring range, so it was selected for further research.

### 3.5. Dynamic Response

**Figure 3:**
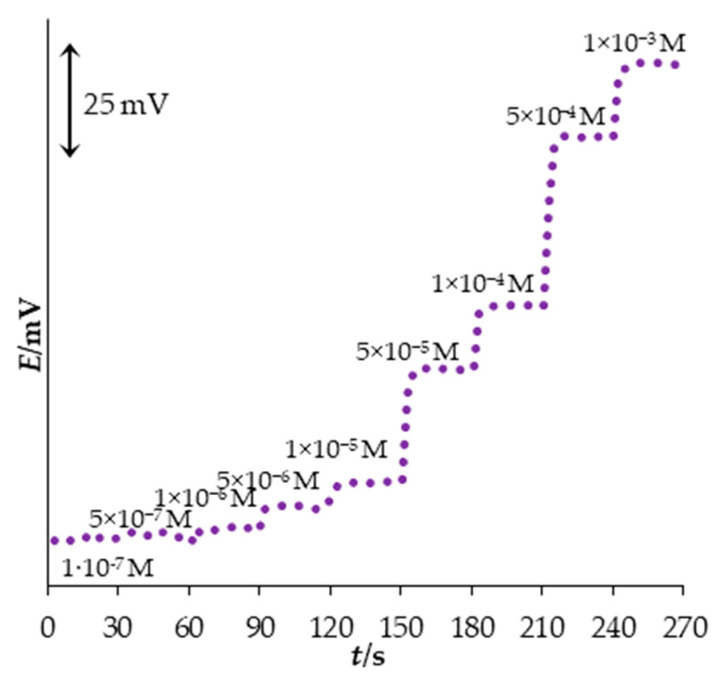
Dynamic response of the new PM sensor.

After immersion of the sensor in the analyte solution, it takes some time for the potential to stabilize. The time that is taken for the sensor to reach 90% of the final value of the potential after a sudden increase in the analyte concentration represents the dynamic response time of the sensor [40]. The response time of the new PM sensor was determined by measuring its potential in an analyte solution where PM concentration was suddenly changed every 30 s in a concentration range between 1.0 × 10^−7^ M and 1.0 × 10^−3^ M. The results are presented in Figure 3, where it can be seen that the new sensor has a very fast response. Its average response time was only 6 s.

### 3.6. Signal Drift

ISEs suffer from the change of the potential over time (signal drift). It can affect the analytical performance of the sensor. Considering the above, the signal drift of the new PM sensor was measured in PM solution (*c* = 4.0 × 10^−3^ M) for five hours. Based on the calculation obtained using the linear regression analysis, it can be described by the equation E (mV) = −0.0006·t(s) + 179.61. The signal drift for the new PM sensor amounted to -1.2 mV/hour.

### 3.7. The Influence of the pH

**Figure 4:**
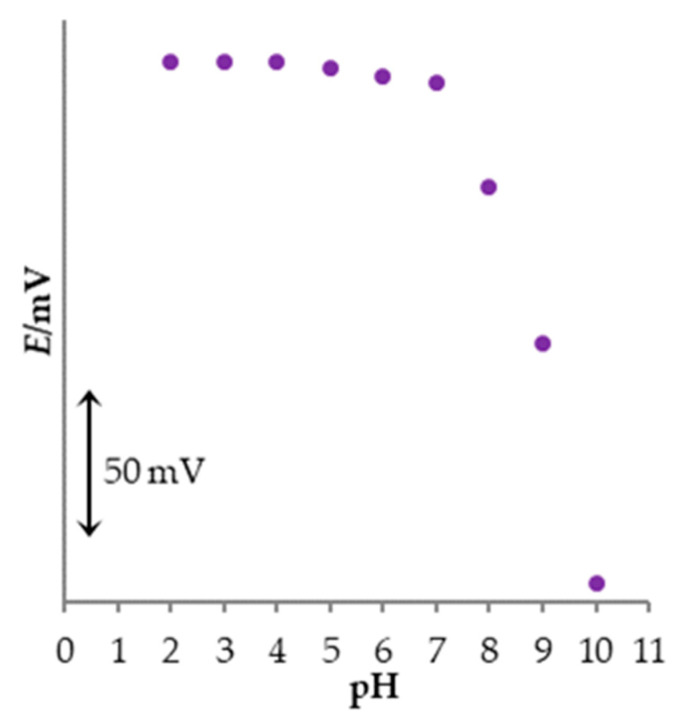
The influence of the pH value on the response of the new PM sensor.

The effect of pH value on the potentiometric response of the new PM sensor was examined in the pH range between 2 and 10 in PM solution (*c* = 4.0 × 10^−3^ M). The results are presented in Figure 4. It can be concluded that the measuring pH range of the new PM sensor is between 2 and 7, due to the potential stability. At pH 8 or above, a significant potential decrease was detected because of the decrease of the ionic form of PM in test solution. It is a result of the hydrolysis reaction and consequently, formation of the free promethazine base [19,20,41].

### 3.8. The Selectivity

Successful determination of the analyte mostly depends on the selectivity of the sensor for the analyte. For that purpose, potentiometric selectivity coefficients ($K_{A,\;B}^{pot}$) were determined using the fixed interference method [39] and Solver (Microsoft Excel) for mathematical adjustment of the Nikolskii–Eisenman equation (Equation (3)) to experimental data.

(3) $$E=E^{{}^{\circ}}+\frac{2.303RT}{z_{A}F}\log\left[a_{A}+\overset{N}{\underset{B=1}{\sum }}K_{A,B}^{pot}a_{B}^{\frac{z_{A}}{z_{B}}}\;\right]$$

In Equation (3), $a_{A}$ and $z_{A}$ represent activity and charge of the analyte ion, respectively, and $a_{B}$ and $z_{B}$ represent activity and charge of the interfering ion, respectively.

The experiments were performed in solutions containing potential interferent (*c* = 1.0 × 10^−2^ M) and PM (its concentration varied between 2.0 × 10^−6^ M and 2.0 × 10^−3^ M). The potential interferents were chosen due to their presence in composition of pharmaceutical products and body fluids. The calculated $K_{A,\;B}^{pot}$ values are presented in Table 4. It can be seen that the new PM sensor is selective for PM, so it could be applicable for PM determination in real samples. Although results suggest that the new PM sensor could be applicable for PM determination in pharmaceutical products and body fluids, in this study, the new sensor was used for PM determination in pharmaceutical products.

### 3.9. Determination of PM

A study on the applicability of the new sensor for PM determination was performed using potentiometric titration and the Gran method [42]. Two commercial drops containing PM as the active pharmaceutical ingredient in a concentration of 20 mg/mL were used as test solutions. The solutions were prepared by dissolving drops in distilled water. For the titration method, NaTPB solution (*c* = 4.0 × 10^−3^ M) was used as the titrant. For the Gran method, increments of known concentration (*c* = 2.0 × 10^−3^ M) of PM were added to the solution eight times. After each addition, the potential was measured. Using Equation (4) and constructing the plot of $10^{\frac{E_{1}-E_{0}}{S}}\left(V_{0}+V_{s}\right)$ against $c_{s}V_{s}$, the linear graph was obtained. The negative x-intercept represents the negative amount of PM in the initial sample. Knowing the amount of PM, it was easy to calculate its concentration.

(4) $$10^{\frac{E_{1}-E_{0}}{S}}\left(V_{0}+V_{s}\right)=V_{0}+\frac{1}{c_{x}}c_{s}V_{s}$$

In Equation (4), $E_{1}$ and $E_{0}$ represent measured potential after each addition and before addition, respectively; *V*_0_ and *V*_s_ represent the volume of the solution before standard addition and volume of each standard addition, respectively; *c*_x_ and *c*_s_ represent PM concentration before standard addition and concentration of each standard addition solution, respectively.

The comparison of the results with the manufacturer’s claimed values was used to check the accuracy of PM determination. The results are presented in Table 5.

It can be seen that all results are acceptable, and there is no influence of the matrix components on the accuracy of PM determination. However, the results obtained using potentiometric titration are more precise. Additionally, recoveries for PM determination using potentiometric titration are in the range ±5%, while recoveries for PM determination using the Gran method are in the range ±10%. It was expected because the Gran method is the direct potentiometry method.

### 3.10. The Lifetime

The lifetime of the sensor is a time in which the sensor has constant analytical characteristics and can be used for accurate determination of the analyte. In order to determine the lifetime of the new PM sensor, every day before measurements, the calibration in a concentration range between 5.0 × 10^−6^ M and 5.0 × 10^−3^ M was performed. With daily measurements, the lifetime of the new PM sensor is at least four months, without significant deviations in the performance of the sensor.

## 4. Conclusions

The new potentiometric solid-contact sensor, with an ionophore based on functionalized MWCNTs and PM ions, was developed for PM determination. The membrane of the sensor was optimized using five different plasticizers and different content of sensing material (ionophore) in the membrane. The sensor with NPPE and 4% of sensing material was chosen for further characterization due to theoretical calculations and the best analytical performances. It had a Nernstian slope, low LOD (1.5 × 10^−7^ M), a measuring range between 6.2 × 10^−7^ M and 5.0 × 10^−3^ M, good selectivity, and very fast response. The introduction of MWCNTs in the membrane of the sensor resulted in its low signal drift and good stability. The applicability of the new PM sensor was demonstrated using the Gran method and potentiometric titration for accurate PM determination in a pure aqueous solution of PM and pharmaceutical products. Due to the results of the selectivity investigation, the new sensor also has the potential for PM determination in biological fluids.

## Figures and Tables

**Table 1:** The logP, logS, and HSP for plasticizers and PM.

	R_a_	δd	δp	δh	logP	logS
PM	-	19.52	6.64	5.33	4.27	−1.99
NPPE	1.10	19.87	7.39	5.01	3.23	−2.68
DBP	4.37	17.57	6.7	3.28	4.93	−3.64
*o*-NPOE	5.18	17.71	10.21	4.21	4.95	−4.32
DOP	6.15	16.91	5.15	2.37	8.79	−5.75
DS	7.54	16.23	3.11	4.03	6.82	−4.94

**Table 2:** Statistics of the response characteristics of the PM sensors with different plasticizers, to PM ^1^.

Plasticizer	Content of the Sensor Material (%)	Slope (mV/Decade of Activity)	Standard Error	Correl. Coeff. (R^2^)	LOD (M)	Useful Conc. Range (M)
DOP	2	51.0 ± 1.4	3.9	0.9947	1.7 × 10^−6^	2.4 × 10^−6^–5.0 × 10^−3^
DS	2	51.2 ± 1.3	3.7	0.9953	8.6 × 10^−7^	1.7 × 10^−6^–5.0 × 10^−3^
*o*-NPOE	2	52.1 ± 0.8	1.8	0.9987	1.7 × 10^−6^	4.6 × 10^−6^–5.0 × 10^−3^
DBP	2	53.8 ± 0.5	1.1	0.9995	1.2 × 10^−6^	3.3 × 10^−6^–5.0 × 10^−3^
NPPE	2	55.8 ± 0.6	1.5	0.9992	1.2 × 10^−6^	1.7 × 10^−6^–5.0 × 10^−3^

^1^ Average of 5 determinations ± confidence limits (*p* = 0.95).

**Table 3:** Statistics of the response characteristics of the PM sensors with different content of sensor material, to PM ^1^.

Plasticizer	Content of the Sensor Material (%)	Slope (mV/Decade of Activity)	Standard Error	Correl. Coeff. (R^2^)	LOD (M)	Useful Conc. Range (M)
NPPE	2	55.8 ± 0.6	1.5	0.9992	1.2 × 10^−6^	1.7 × 10^−6^–5.0 × 10^−3^
NPPE	4	59.4 ± 1.1	3.8	0.9973	1.5 × 10^−7^	6.2 × 10^−7^–5.0 × 10^−3^
NPPE	6	52.1 ± 1.1	3.5	0.9966	4.5 × 10^−7^	8.6 × 10^−7^–5.0 × 10^−3^

^1^ Average of 5 determinations ± confidence limits (*p* = 0.95).

**Table 4:** Potentiometric selectivity coefficients of the new PM sensor.

Interference	$K_{A,\;B}^{pot}$
Ammonium	8.16 × 10^−4^
Sodium	3.66 × 10^−4^
Calcium	6.99 × 10^−5^
Magnesium	2.21 × 10^−4^
Ascorbic acid	6.11 × 10^−3^
Manganese	6.84 × 10^−5^
Potassium	4.98 × 10^−4^
Copper	2.08 × 10^−4^
Zinc	6.53 × 10^−5^
Lithium	2.24 × 10^−3^
Iron (III)	1.20 × 10^−5^
Cobalt	2.36 × 10^−4^
Naproxen	1.28 × 10^−3^
Glycine	3.09 × 10^−3^
Caffeine	2.06 × 10^−4^

**Table 5:** Results of the PM determination in pure aqueous solution and pharmaceutical preparations, obtained using potentiometric titration and the Gran method ^1^.

Sample	Pure PM Solution		Atosil Drops Solution		Promethazin-Neuraxpharm Drops Solution	
Method	Titration Method	Gran Method	Titration Method	Gran Method	Titration Method	Gran Method
Claimed value (M)	4.00 × 10^−3^	1.00 × 10^−4^	4.00 × 10^−3^	1.00 × 10^−4^	4.00 × 10^−3^	1.00 × 10^−4^
PM found (M) ± RSD (%)	4.09 × 10^−3^ ± 0.3	1.07 × 10^−4^ ± 4.7	4.02 × 10^−3^ ± 0.2	1.09 × 10^−4^ ± 5.1	4.13 × 10^−3^ ± 0.2	1.03 × 10^−4^ ± 6.1
Recovery (%)	102.3	107.0	100.5	109.0	103.3	103.0

^1^ Average of 3 determinations.

## Data Availability

The data presented in this study are available on request.
